# Supplementary material for: Significance of TERT Genetic Alterations and Telomere Length in Hepatocellular Carcinoma
Source: Cancers (Basel). 2021 Apr 30;13(9):2160. doi: 10.3390/cancers13092160 (PMC8125722; doi:10.3390/cancers13092160)
Supplement: Supplementary file 1 [file cancers-13-02160-s001.zip › cancers-1209515-supplementary-Corrected.pdf]

*Supplementary Materials*

# Significance of *TERT* Genetic Alterations and Telomere Length in Hepatocellular Carcinoma

**Jeong-Won Jang, Jin-Seoub Kim, Hye-Seon Kim, Kwon-Yong Tak, Soon-Kyu Lee, Hee-Chul Nam, Pil-Soo Sung, Chang-Min Kim, Jin-Young Park, Si-Hyun Bae, Jong-Young Choi and Seung-Kew Yoon**

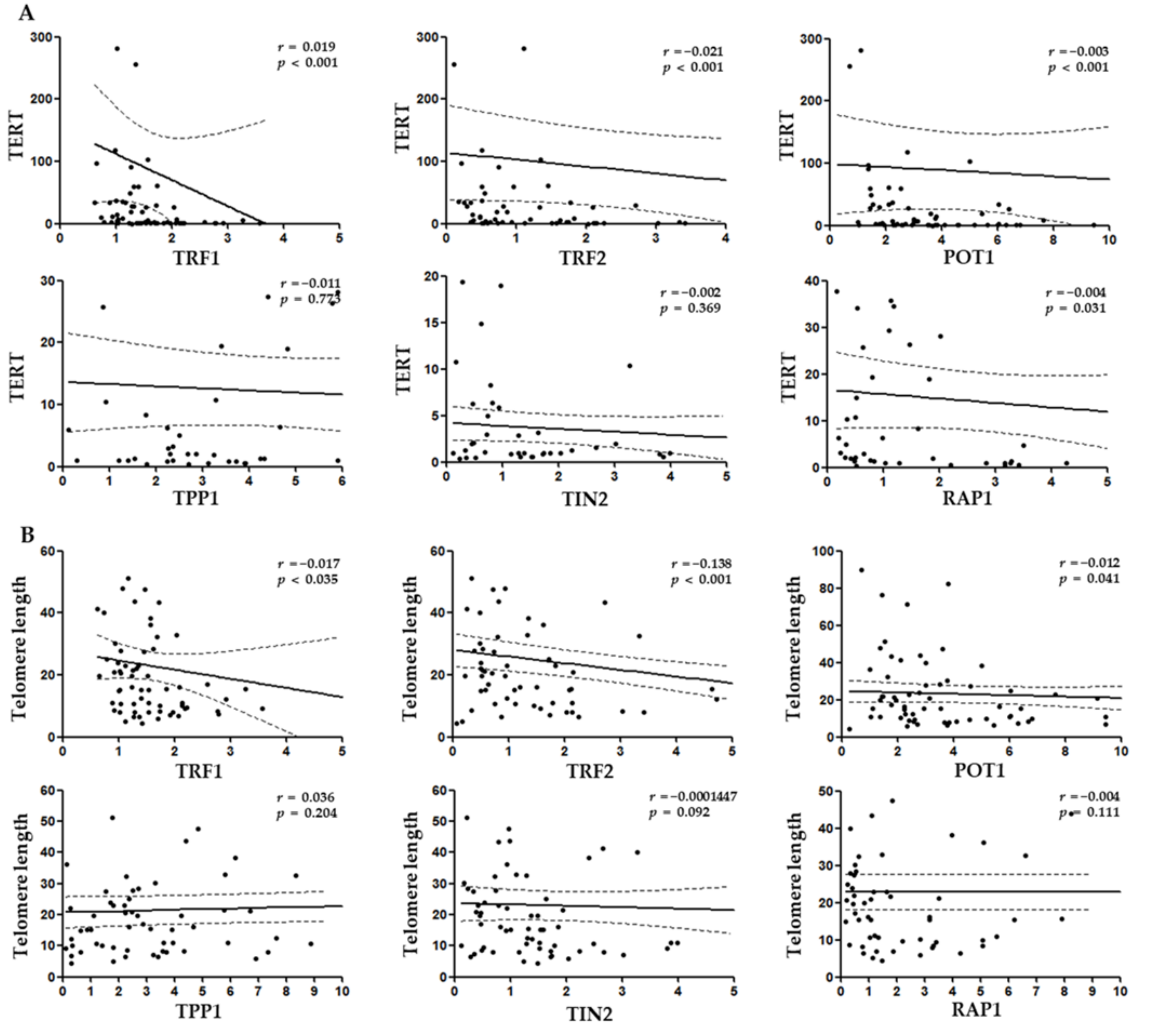


**Figure S1.** Correlation between the expression statuses of the shelterin complex protein, *TERT* expression, and telomere length. (**A**) Correlation between shelterin complex protein and TERT in overall HCC patients. (**B**) Correlation between shelterin complex protein and telomere length in overall HCC patients. TERT, telomerase reverse transcriptase; HCC, hepatocellular carcinoma; TRF, telomeric repeat-binding factors; POT1, protection of telomeres 1; TPP1, POT1-TIN2 organizing protein; TIN2, TRF1 and TRF2 interacting nuclear protein 2; RAP1, repressor/activator protein 1.

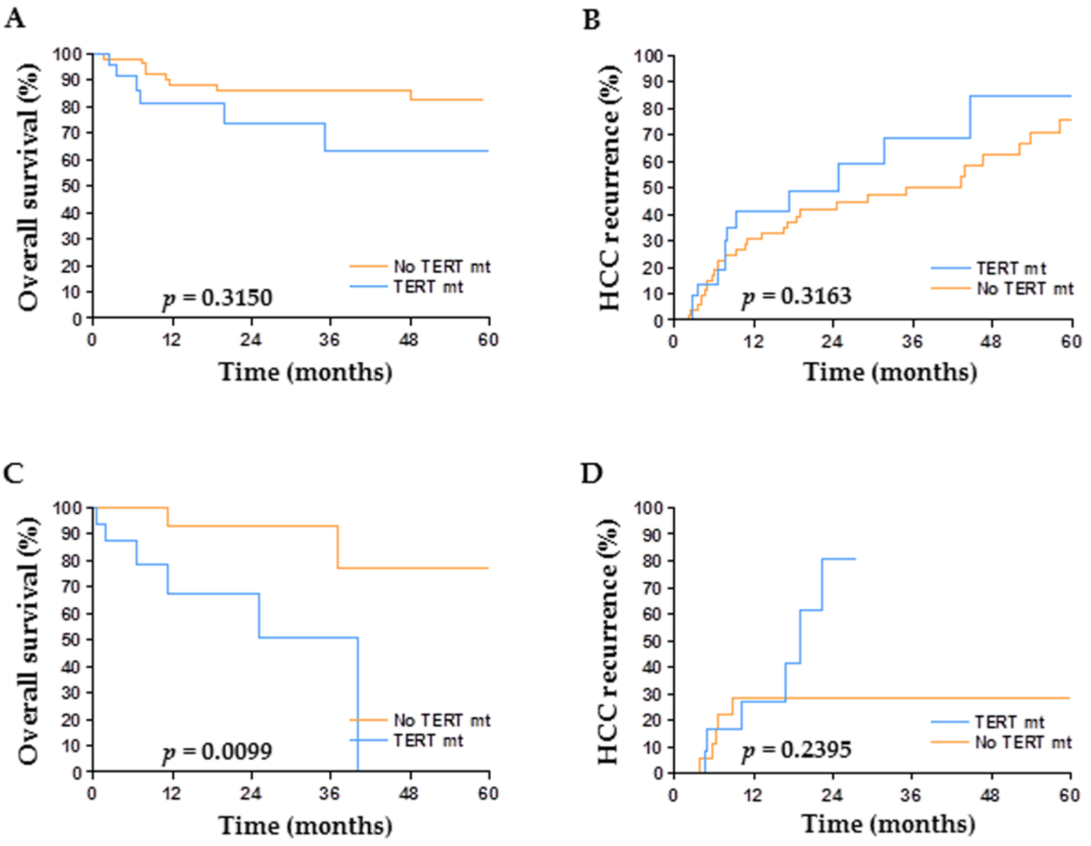


**Figure S2.** Relationship between the *TERT* promoter mutation and the outcome of patients undergoing surgical resection according to the cause of HCC. Overall survival and HCC recurrence. (**A**,**B**) Overall survival and HCC recurrence in patients with HBV-related HCC. (**C**,**D**) Overall survival and HCC recurrence in patients with non-HBV-related HCC. TERT, telomerase reverse transcriptase; mt, mutation.

**Table S1.** Functional interactions between the eight *TERT*-interacting genes.

| Interacting Gene | Nature and Function of These Proteins | Quote | Reference |
|---|---|---|---|
| *mTOR* | - mTOR is a member of the phosphatidylinositol 3-kinase-related kinase family of protein kinases.<br>- mTOR functions as a serine/threonine protein kinase that regulates cell growth, cell proliferation, cell motility, cell survival, protein synthesis, autophagy, and transcription. | - TERT inhibits the kinase activity of mTOR complex 1 (mTORC1) in multiple cell lines, resulting in the activation of autophagy under both basal and amino acid-deprived conditions.<br>- hTERT overexpression decreased the kinase activity of mTORC1 as determined by decreased phosphorylation of the mTORC1 target p70S6K (p-T389). | Ali, M.; Devkota, S.; Roh, J.-I.; Lee, J.; Lee, H.-W. J. B.; Telomerase reverse transcriptase induces basal and amino acid starvation-induced autophagy through mTORC1. *Biochem. Biophys. Res. Commun*. 2016, 478 (3), 1198–1204 |
| | | - mTOR regulates telomerase activity at the translational and post-translational level.<br>- mTOR inhibitor rapamycin reduces telomerase activity without changing hTERT mRNA activity.<br>- Correlation between mTOR and hTERT is important for survival and immortality of cancer cells. | Dogan, F.; Avci, C. B. J. G., Correlation between telomerase and mTOR pathway in cancer stem cells. *Gene* 2018, 641, 235–239. |
| | | - TERT protein accumulated in mitochondria from brain tissue of mice that have undergone short-term dietary restriction (DR) and rapamycin treatment. This localization was correlated with lower levels of oxidative stress in these brain mitochondria. Since rapamycin treatment decreases mTOR signaling which is also thought to play an important role for the beneficial effects of DR, the mTOR pathway might be involved in the TERT localization and its effects in brain mitochondria in vivo. | Miwa, S.; Saretzki, G. J. N. r. r., Telomerase and mTOR in the brain: the mitochondria connection. *Neural Regen. Res.* 2017, 12 (3), 358. |
| | | - Telomerase deficient mice with short telomeres (G2-*Terc*−/−) have an hyper-activated mTOR pathway with increased levels of phosphorylated ribosomal S6 protein in liver, skeletal muscle and heart, a target of mTORC1. | Ferrara-Romeo, I.; Martinez, P.; Saraswati, S.; Whittemore, K.; Graña-Castro, O.; Poluha, L. T.; Serrano, R.; Hernandez-Encinas, E.; Blanco-Aparicio, C.; Flores, J. M. J. N. c., et al. The mTOR pathway is necessary for survival of mice with short telomeres. *Nat. Commun.* 2020, 11 (1), 1–17. |
| | | - The mTOR signaling pathway impinges on the mitochondrial localisation of TERT protein, which might, in turn, contribute to the protection of the brain by DR or rapamycin against age-associated mitochondrial ROS increase and cognitive decline. | Miwa, S.; Czapiewski, R.; Wan, T.; Bell, A.; Hill, K. N.; von Zglinicki, T.; Saretzki, G. J. A., Decreased mTOR signalling reduces mitochondrial ROS in brain via accumulation of the telomerase protein TERT within mitochondria. *Aging (Albany N.Y.)* 2016, 8 (10), 2551. |
| *AKT* | **-** Akt is a serine/threonine-specific protein kinase that plays a key role in multiple cellular processes such as glucose metabolism, apoptosis, cell proliferation, transcription, and cell migration. | - AKT activation by epidermal growth factor increases hTERT expression and telomerase activity. | Sasaki, T.; Kuniyasu, H.; Luo, Y.; Kitayoshi, M.; Tanabe, E.; Kato, D.; Shinya, S.; Fujii, K.; Ohmori, H.; Yamashita, Y. J. P., et al. AKT activation and telomerase reverse transcriptase expression are concurrently associated with prognosis of gastric cancer. *Pathobiology* 2014, 81 (1), 36–41. |
| | | - The catalytic subunit, telomerase reverse transcriptase (TERT), is regulated by interaction with the 90 kDa heat shock protein (HSP90) and by Akt-dependent phosphorylation. The HSP90 and Akt physically interact with TERT. Likewise, the induction of Akt dephosphorylation by protein phosphatase 2A (PP2A) reduced telomerase activity. | Haendeler, J.; Hoffmann, J.; Rahman, S.; Zeiher, A. M.; Dimmeler, S. J. F. l., Regulation of telomerase activity and anti-apoptotic function by protein–protein interaction and phosphorylation. *FEBS Lett*. 2003, 536 (1-3), 180–186. |
| | | - TERT mediates AKT activity by regulating PTEN expression in HCC cells. | Yu, J.; Yuan, X.; Sjöholm, L.; Liu, T.; Kong, F.; Ekström, T. J.; Björkholm, M.; Xu, D. J. C. l., Telomerase reverse transcriptase regulates DNMT3B expression/aberrant DNA methylation phenotype and AKT activation in hepatocellular carcinoma. *Cancer Lett*. 2018, 434, 33–41. |

| | | | |
|---|---|---|---|
| *YWHAZ* | - YWHAZ protein encoded by this gene is a member of the 14-3-3 protein family and a central hub protein for many signal transduction pathways. | - To evaluate the oncogenic potentials of *YWHAZ*, the study researchers established the noncancerous HaCaT cell line with ectopically transfected *YWHAZ*. Previously, *HRAS*, *BCL2*, *MYC*, *TERT*, and *CCND1* had been overexpressed ectopically in HaCaT, an immortalized skin keratinocyte line. | Lin, M.; Morrison, C. D.; Jones, S.; Mohamed, N.; Bacher, J.; Plass, C. J. I. j. o. c., Copy number gain and oncogenic activity of YWHAZ/14-3-3ζ in head and neck squamous cell carcinoma. *Int. J. Cancer* 2009, 125 (3), 603–611. |
| *YWHAZ*<br>*YWHAQ* | - YWHAQ gene product belongs to the 14-3-3 family of proteins that mediate signal transduction by binding to phosphoserine-containing proteins. | - YWHAQ, YWHAE, and YWHAZ genes (tyrosine 3-monooxygenase/tryptophan 5-monooxygenase activation protein theta, epsilon, and zeta, respectively) belong to the 14-3-3 family that is involved in metabolism, protein trafficking, signal transduction, evasion of apoptosis, cell cycle regulation, cell death, and mitogenesis.<br>- Members of the 14-3-3 protein family antagonize the activity of proteins that promote cell death and senescence, such as Bad, Bim, Bax, and SFN.<br>- This family of proteins are hTERT-binding partners and its interaction is required for efficient accumulation of hTERT in the nucleus. | de la Guardia, R. D.; Catalina, P.; Panero, J.; Elosua, C.; Pulgarin, A.; López, M. B.; Ayllón, V.; Ligero, G.; Slavutsky, I.; Leone, P. E. J. J. o. c.; et al. Expression profile of telomere-associated genes in multiple myeloma. *J. Cell. Mol. Med.* 2012, 16 (12), 3009–3021. |
| *CCT5* | - CCT5 gene encodes a molecular chaperone that is member of the TRiC complex. This complex consists of two identical stacked rings, each containing eight different proteins. | No specific remarks | |
| *TUBA1B* | - TUBA1B has been shown to interact with PIK3R1. Antibodies against tubulin alpha 1b can be used as markers for microtubules and spindles. | No specific remarks | |
| *RPS6KB1* | - RPS6KB1 regulates insulin action on glucose metabolism in skeletal muscle and estrogen receptor alpha (ERalpha) by phosphorylating it on serine 167, leading to transcriptional activation of ERalpha. | No specific remarks | |

| **Gene** | **Interaction Gene** | **Quote** | **Reference** |
|---|---|---|---|
| *mTOR* | RPS6KB1<br>(=S6K1) | S6K1 and S6K2 silencing leads to a compensatory transcriptional upregulation of mTORC1, as well as mTORC2. | Karlsson, E.; Magić, I.; Bostner, J.; Dyrager, C.; Lysholm, F.; Hallbeck, A.-L.; Stål, O.; Lundström, P. J. P. o., Revealing different roles of the mTOR-targets S6K1 and S6K2 in breast cancer by expression profiling and structural analysis. *PLOS ONE* 2015, 10 (12), e0145013. |
| *mTOR* | CCT5 | - CCT contributes to mTORC assembly and signaling. | Cuéllar, J.; Ludlam, W. G.; Tensmeyer, N. C.; Aoba, T.; Dhavale, M.; Santiago, C.; Bueno-Carrasco, M. T.; Mann, M. J.; Plimpton, R. L.; Makaju, A. J. N. c., et al. Structural and functional analysis of the role of the chaperonin CCT in mTOR complex assembly. *Nat. Commun.* 2019, 10 (1), 1–14. |
| *AKT* | YWHAZ | - High YWHAZ expression was observed to promote cell proliferation and inhibit apoptosis through AKT targeting in AML. | Gan, Y.; Ye, F.; He, X.-X. J. J. o. C., The role of YWHAZ in cancer: A maze of opportunities and challenges. J. Cancer 2020, 11 (8), 2252. |
| *AKT* | YWHAQ | - In HEK293T cells, AKT promotes G2/M cell-cycle progression by inducing phophorylation-dependent YWHAQ binding and cytoplasmic localization of WEE 1 kinase. | Feng, C.; Yu, A.; Liu, Y.; Zhang, J.; Zong, Z.; Su, W.; Zhang, Z.; Yu, D.; Sun, Q.-Y.; Yu, B. J. B. o. r., Involvement of protein kinase B/AKT in early development of mouse fertilized eggs. *Biol. Reprod.* 2007, 77 (3), 560–568. |

**Table S2.** Correlation between the expression statuses of the shelterin complex protein, *TERT* expression, and telomere length.

| Shelterin Genes | Overall ($n$ = 68) | | | | Non-Tumor ($n$ = 22) | | | | Tumor ($n$ = 46) | | | |
|---|---|---|---|---|---|---|---|---|---|---|---|---|
| | Telomere Length | | TERT Expression | | Telomere Length | | TERT Expression | | Telomere Length | | TERT Expression | |
| | Rho * | *P* | Rho * | *P* | Rho * | *P* | Rho * | *P* | Rho * | *P* | Rho * | *P* |
| *TRF1* | −0.256 * | 0.035 * | −0.454 ** | 0.000 *** | 0.010 | 0.963 | −0.297 | 0.179 | 0.067 | 0.658 | −0.166 | 0.270 |
| *TRF2* | −0.469 ** | 0.000 *** | −0.527 ** | 0.000 *** | −0.256 * | 0.250 | 0.139 | 0.539 | −0.113 | 0.456 | −0.067 | 0.657 |
| *POT1* | −0.249 * | 0.041 * | −0.398 ** | 0.001 ** | 0.001 | 0.996 | −0.226 | 0.311 | −0.085 | 0.575 | −0.341 * | 0.020 * |
| *TPP1* | 0.156 | 0.204 | −0.036 | 0.773 | −0.176 | 0.432 | −0.113 | 0.617 | 0.302 | 0.041 | −0.061 | 0.685 |
| *TIN2* | −0.206 | 0.092 | −0.111 | 0.369 | −0.093 | 0.618 | 0.153 | 0.497 | −0.038 | 0.802 | 0.269 | 0.070 |
| *RAP1* | −0.195 | 0.111 | −0.262 * | 0.031 * | −0.113 | 0.618 | −0.435 * | 0.043 * | 0.183 | 0.223 | 0.363* | 0.013 * |

**, Correlation is significant at the 0.01 level (2-tailed); *, Correlation is significant at the 0.05 level (2-tailed); $p$-value indicates $p < 0.05$ *, $p < 0.01$ **, $p < 0.001$ ***.

**Table S3.** Primer and probe design for gene expression.

| Primers for Gene Expression | | |
|---|---|---|
| Primer | F/R/P | Sequence |
| CCT5 | Forward | 5′-CATGGCCCTCTCTGAAAACAG-3′ |
| | Reverse | 5′-GAGCAGGGTTCATCTCCTTCAC-3′ |
| | Probe | 5′-TCCCATCCAGACTATGACCGAAGTCCG-3′ |
| TUBA1B | Forward | 5′-TGGTGTGGATTCTGTTGAAGGA-3′ |
| | Reverse | 5′-CTGAAATTCTGGGAGCATGACA-3′ |
| | Probe | 5′-CTAATTATCCATTCCTTTTGGCCCTGCAGC-3′ |
| MTOR | Forward | 5′-AGGCCGCATTGTCTCTATCAA-3′ |
| | Reverse | 5′-GCAGTAAATGCAGGTAGTCATCCA-3′ |
| | Probe | 5′-TGCAATCCAGCTGTTTGGCGCC-3′ |
| RPS6KB1 | Forward | 5′-CCGAACTCTGGGCCATACA-3′ |
| | Reverse | 5′-TTGCAGGATGCTCACACATCTC-3′ |
| | Probe | 5′-CAAACGGCCAGAGCACCTGCGT-3′ |
| AKT1 | Forward | 5′-TCTCGGGTGCATTTGAGAGAA-3′ |
| | Reverse | 5′-ACAGCACAAAAACGTCTTTCCA-3′ |
| | Probe | 5′-CCACGCTGTCCTCTCGAGCCCA-3′ |
| YWHAZ | Forward | 5′-CCAGTGTAAAACTGCTTCCATGTC-3′ |
| | Reverse | 5′-TACACCTGTGACTGGAACCAATG-3 |
| | Probe | 5′-AACTGCCTACATACTGGTTTGTCCTGGCG-3′ |
| YWHAQ | Forward | 5′-CCTGGAGGGTCATCTCTAGCAT-3′ |
| | Reverse | 5′-ACTTTCTCCCGATAGTCCTTAATCAG-3′ |
| | Probe | 5′-AGCAGAAGACCGACACCTCCGACAAG-3′ |

**Table S4.** PCR conditions with the primer sequences for the shelterin complex.

| Primers for Shelterin Complex | | |
|---|---|---|
| Primer | F/R | Sequence |
| TRF1 | Forward | 5′-TCTCTCTTTGCCGAGCTTTCC-3′ |
| | Reverse | 5′-ACTGGCAAGCTGTTAGACTGGAT-3′ |
| TRF2 | Forward | 5′-GGTACGGGGACTTCAGACAG-3′ |
| | Reverse | 5′-CGCGACAGACACTGCATAAC-3′ |
| POT1 | Forward | 5′-TTCCACTAAAGAGCAGGCAA-3′ |
| | Reverse | 5′-TGAAGTTCTTTAAGCCCCCA-3′ |
| TPP1 | Forward | 5′-TCACCAGATCAGCCACATTC-3′ |
| | Reverse | 5′-TGGAAAGACTCTCGGAGCTG-3′ |
| TIN2 | Forward | 5′-TGCTTTCAGTGGCTCTTCTG-3′ |
| | Reverse | 5′-TTTACCAGCAGGTGAAGCAG-3′ |
| RAP1 | Forward | 5′-TCTTCTTCAGGCAAATCTGGA-3′ |
| | Reverse | 5′-CCTCCTCCCAGAAGCTCAA-3′ |
| GAPDH | Forward | 5′-GAGTCAACGGATTTGGTCGT-3′ |
| | Reverse | 5′-TTGATTTTTGGAGGGATCTCG-3′ |
| Conditions | | 95 °C for 10 min, 94 °C for 15 s, 60 °C for 60 s for 40 cycles |
